# Effects of Endotoxaemia on Protein Metabolism in Rat Fast-Twitch Skeletal Muscle and Myocardium

**DOI:** 10.1371/journal.pone.0006945

**Published:** 2009-09-14

**Authors:** Andrew J. Murton, Nima Alamdari, Sheila M. Gardiner, Dumitru Constantin-Teodosiu, Robert Layfield, Terence Bennett, Paul L. Greenhaff

**Affiliations:** Centre for Integrated Systems Biology and Medicine, School of Biomedical Sciences, Queen's Medical Centre, University of Nottingham, Nottingham, United Kingdom; University Hospital Vall d'Hebron, Spain

## Abstract

**Background:**

It is unclear if the rat myocardium undergoes the same rapid reductions in protein content that are classically observed in fast-twitch skeletal muscle during endotoxaemia.

**Methodology/Principal Findings:**

To investigate this further, and to determine if there is any divergence in the response of skeletal muscle and myocardium in the mechanisms that are thought to be largely responsible for eliciting changes in protein content, Sprague Dawley rats were implanted with vascular catheters and administered lipopolysaccharide (LPS; 150 µg kg^−1^ h^−1^) intravenously for 2 h, 6 h or 24 h (saline administered control animals were also included), after which the extensor digitorum longus (EDL) and myocardium were removed under terminal anaesthesia. The protein-to-DNA ratio, a marker of protein content, was significantly reduced in the EDL following 24 h LPS administration (23%; P<0.05), but was no different from controls in the myocardium. At the same time point, a significant increase in MAFbx/atrogin-1 and MuRF1 mRNA (3.7±0.7- and 19.5±1.9-fold increase vs. controls, respectively; P<0.05), in addition to protein levels of α1-3, 5–7 subunits of the 20S proteasome, were observed in EDL but not myocardium. In contrast, elevations in phosphorylation of p70 S6K residues Thr^421^/Ser^424^, and 4E-BP1 residues Thr^37^/Thr^46^ (P<0.05), consistent with an elevation in translation initiation, were seen exclusively in the myocardium of LPS-treated animals.

**Conclusions/Significance:**

In summary, these findings suggest that the myocardium does not undergo the same catabolic response as skeletal muscle during early endotoxaemia, partly due to the absence of transcriptional and signalling events in the myocardium typically associated with increased muscle proteolysis and the suppression of protein synthesis.

## Introduction

A well known consequence of sepsis is the rapid and debilitating loss of skeletal muscle mass, which appears the result of a coordinated reduction of muscle protein synthesis together with an enhancement of proteasomal mediated muscle protein breakdown [1], [2]. However, the effects of sepsis on the mechanisms that regulate protein turnover in the myocardium are, in comparison, poorly understood.

We have recently shown the induction of lipopolysaccharide (LPS)-induced endotoxaemia in rodents to result in the reduction of total AKT protein levels, decreased cytosolic Foxo1 and Foxo3 phosphorylation, and increased levels of MAFbx/atrogin-1 protein and MuRF1 mRNA in fast-twitch skeletal muscle [3], where MAFbx/atrogin-1 and MuRF1 are considered important in the development of skeletal muscle proteolysis during catabolic events [4]–[6]. These *in vivo* findings are important because, when viewed in conjunction with similar observations in C2C12 cells [7], [8], they suggest AKT as a regulator of both protein translation initiation and ubiquitin-proteasome (UP) mediated muscle proteolysis. Indeed, AKT is well known as an upstream regulator of translation initiation, where increases in AKT activity lead to increases in both eIF2B guanidine exchange factor activity and eIF4F assembly, via the phosphorylation and subsequent inhibition of GSK3β and 4E-BP1, respectively [9], processes that are pivotal to the correct formation of a functioning ribosomal initiation complex for translation initiation to occur. Furthermore, when activated, AKT can phosphorylate the forkhead Foxo family of transcription factors, leading to their transportation away from the nucleus and into the cytoplasm where they are inactive [7], [8]. However, when present in the nucleus, Foxo1, Foxo3 and Foxo4 can induce the transcription of the two muscle-specific ubiquitin ligases, MAFbx/atrogin-1 and MuRF1, which are thought responsible for the specific targeting of proteins for subsequent degradation by the proteasome [7], [8].

Interestingly, while numerous studies have focused on skeletal muscle when considering the endotoxaemia-induced derangements that occur in the pathways that regulate translation initiation [10]–[12] and UP-mediated proteolysis [13], [14], few have examined if these pathways are likewise perturbed in the myocardium. Moreover, from the limited evidence available, it is currently not possible to discern if the rat myocardium undergoes the same rapid reduction in protein content classically observed in rodent fast-twitch skeletal muscle. Conflicting reports detailing both a decline [15] and the maintenance [16], [17] of myocardial protein content following experimentally induced endotoxaemia have been described. Thus, we sought to determine if an i.v., non-lethal dose of bacterial LPS, administered to conscious rats, resulted in a change in myocardial protein content, and examined in the same tissue, transcriptional and signalling events that are typically associated with endotoxaemia-induced protein loss in fast-twitch skeletal muscle [18]. An attempt was also made to relate any observed differences between myocardial and skeletal muscle tissue to localised levels of the two cytokines, tumor necrosis factor-alpha (TNFα) and interleukin-6 (IL-6), as they have, in part, been implicated in eliciting reductions in muscle protein content [19], [20]. Here we show that while significant declines in skeletal muscle protein content are observed in response to LPS infusion, myocardial protein content is maintained at normal levels. Furthermore, we present evidence to suggest that this is in part due to a differential response to endotoxin in the processes that govern UP-mediated degradation and translation initiation in these two tissues.

## Materials and Methods

### Ethics Statement

All procedures involving live animals, as detailed below, were approved by the University of Nottingham Ethical Review Committee and were performed under U.K. Home Office Project and Personal License authority.

### Animal preparation and experimental protocol

Sixty four male Sprague-Dawley rats (380–480 g; Charles River, Margate, UK) were anesthetised using fentanyl (Janssen-Cilag, High Wycombe, UK) and medetomidine (Domitor, Pfizer, Sandwich, UK; 300 µg·kg^−1^ of each i.p.) and had intravenous (right jugular vein) catheters implanted for the administration of substances. In animals designated for 24 h LPS treatment (see below) catheters were also implanted in the distal abdominal aorta (via the ventral caudal artery) for monitoring of blood pressure (BP) and heart rate (HR). We have previously found saline administration, as used in control animals, to have no significant effect on baseline cardiovascular status over 24 h [21], and therefore the BP and HR of control animals were not monitored. Anaesthesia was reversed with atipamezole (Antisedan, Pfizer, Sandwich, UK) and nalbuphine (Bristol-Myers Squibb, Uxbridge, UK; 1 mg·kg^−1^ of each s.c.), with the latter also providing analgesia. Rats were allowed to recover overnight in their home cages and were given free access to both food and water. A counter-balanced tether system connected to a harness and fitted to the rat carried the catheters, and allowed relatively unrestricted movement within the home cage. Double channel swivel systems were constructed according to [22] which enabled the continuous administration of saline or LPS to the unrestrained animals.

Prepared rats were divided into 6 groups; 3 groups were infused with sterile saline (0.4 ml h^−1^) for either 2 h (n = 8), 6 h (n = 7) or 24 h (n = 8), and the remaining 3 groups received a continuous infusion of LPS (*E. coli*, serotype 0127:B8, Sigma, Poole, UK; dissolved in sterile, isotonic saline; 150 µg kg^−1^ h^−1^) for either 2 h (n = 8), 6 h (n = 8) or 24 h (n = 6). At the specified time after onset of saline or LPS infusion, animals were terminally anesthetised (i.v. sodium pentobarbital, Sagatal, Rhône Mérieux, Harlow, UK). The EDL muscle was removed from the left hind-limb during anaesthesia, and was immediately frozen and stored in liquid nitrogen. The myocardium was then rapidly removed and freeze-clamped (whilst still beating) with liquid nitrogen-cooled aluminium tongs, and subsequently transferred to liquid nitrogen for storage. Two 24 h LPS-infused rats and one 6 h saline-infused rat stopped breathing before removal of tissues was complete, and were excluded from the study. To examine muscle signalling events associated with early endotoxaemia, 16 additional animals underwent the procedures described above and received either LPS (n = 8) or saline (n = 8) i.v. for a 24 h period. This sole time-point was selected as previous work emanating from our laboratory had shown 24 h LPS administration was sufficient to result in significant suppression of AKT-mediated signalling events in fast-twitch skeletal muscle [3]. The EDL and myocardium removed from these animals were used exclusively for the investigation of changes in protein and phosphorylation levels of translation initiation signalling events.

### Cardiovascular recordings

Muscle contraction is well known to modulate processes that control muscle mass [23] and we have previously shown LPS administration to result in prolonged and persistent tachycardia [24]. Therefore, to examine any association between the observed tachycardia seen in LPS-treated animals and myocardial transcriptional and signalling events, recordings of heart rate (HR) were made. This was accomplished by using a customised, computer-based system (Haemodynamics Data Acquisition System, University of Limburg, Netherlands) connected to a BP transducer amplifier (Gould model 13-4615-50; Gould Instrument Systems Inc., Valley View, USA), providing both HR and BP recordings. Data were sampled by the Haemodynamics Data Acquisition System every 2 ms, averaged each cardiac cycle, and stored to disc every 5 s. Data were analysed off-line (Datview; University of Maastricht, Netherlands) and measurements were made under resting conditions and at 1 h intervals for the first 6 h after LPS administration and again at 24 h.

### Alkaline soluble protein/DNA ratio

The alkaline soluble protein (ASP)/DNA ratio may be used as a measure of net protein catabolism in skeletal muscle [3], [25], [26]. Approximately 5 mg of muscle tissue was freeze-dried and powdered, with care taken to remove any connective tissue present. ASP and DNA were individually isolated from the powdered muscle using repeated perchloric acid extractions and washings, with the ASP being ultimately extracted by the addition of potassium hydroxide. ASP concentration was determined using the method of Lowry [27], and DNA was quantified using a modification of the diphenyl-amine reaction described by Munro and Fleck [28].

### Real-time quantitative PCR

Total RNA was extracted from approximately 30 mg of frozen muscle using a TRIzol® (Invitrogen) based method that has been described in detail elsewhere [29]. After subsequent extraction, RNA samples were quantified by measuring their absorbance at 260 nm and 280 nm. Reverse transcription was carried out using 1.0 µg of total RNA in a total volume of 30 µl, containing MMLV reverse transcriptase, random hexamer primers, RNase inhibitor N2511, and the four nucleotides dATP, dGTP, dCTP and dUTP (all sourced from Promega, Southampton, UK). After incubation at 42°C for 1 h, freshly synthesised cDNA was diluted four-fold with RNase-free H_2_O.

Six, previously designed, Assay-on-Demand Taqman® primer and probe sets were purchased (Applied Biosystems), MuRF1 (#Rn00590197_m1); MAFbx/atrogin-1 (#Rn00591730_m1); 20S proteasome subunit α1 (PSMA1; #Rn00568675_m1); TNFα (#Rn00562055_m1); IL-6 (#Rn00561420_m1) and the housekeeping gene hydroxymethlybilane synthase (HMBS; #Rn00565886_m1). Each Taqman primer and probe set was validated by performing real-time PCR with a series of 4-fold cDNA template dilutions to obtain standard curves of cycle threshold number (Ct) against log relative concentration (data not shown). All six genes were found to be amplified with equal efficiency allowing the subsequent use of the comparative Ct method (ΔΔCt) for the relative quantification of gene expression. All sample and non-template control reactions were performed in the ABI Prism 7000 Sequence Detection System (Applied Biosystems) in triplicate. The Ct values for each triplicate were averaged and the ΔCt calculated by the subtraction of the corresponding mean Ct value for the normalisation gene HMBS. The expression of HMBS was previously found to be unaffected by LPS-infusion within myocardium and skeletal muscle (data not shown). Real-time PCR was performed using PCR Universal Master Mix (Applied Biosystems) in a MicroAmp 96-well reaction plate. Each well contained 2 µl cDNA template, 12.5 µl PCR Master Mix and 1.25 µl of Assay on Demand primer/probe mix in a reaction volume of 25 µl. Data from the LPS-treatment group was normalised to the average of the saline (control) group for each muscle tissue type and time point.

### Protein levels of 20S proteasome subunits α1-3 & 5–7

Proteins were extracted by homogenisation in 0.8 ml of pH 7.5 Tris-EDTA buffer, and subsequently quantified and normalised using the Lowry method [27]. Protein samples (20 µg) were separated by molecular weight on 5–20% SDS-PAGE gels in 1x Tris/Glycine/SDS buffer (Biorad, Hemel Hempstead, UK) and transferred overnight to nitrocellulose membranes (Amersham Biosciences, Little Chalfont, UK). Equal loading of samples was confirmed by Ponceau S staining of membranes. Membranes were blocked by incubation for 1 h with 4% (w/v) milk protein in tris-buffered saline, and subsequently probed with polyclonal rabbit anti-α1–3, 5–7 subunits of the proteasome (Biomol, Exeter, UK) for 1 h, peroxidase-conjugated donkey anti-rabbit (Amersham Biosciences) for 1 h, and visualised by enhanced chemiluminescence plus (Amersham Biosciences). Thus, the primary antibody cross reacts with the α1 subunit examined by RT-PCR, in addition to other core proteasome subunits.

### Protein and phosphorylation levels of AKT, p70 S6K and 4E-BP1

Proteins were extracted from approximately 30 mg of muscle by homogenisation in 0.8 ml of pH 7.5 homogenisation buffer, consisting of 50 mM Tris-HCL, 1 mM EDTA, 1 mM EGTA, 1% (v/v) Triton® X-100 and 0.1% (v/v) β-mercaptoethanol. In addition, to every 10 ml of homogenisation buffer was added 1 tablet of a protease inhibitor cocktail (Roche, Burgess Hill, UK) and 100 µl of phosphatase inhibitor cocktail II (Sigma). Extracted proteins were subsequently quantified using the method of Lowry et al. [27] and diluted to a concentration of 2 µg/μl with 2x SDS buffer (25% (w/v) glycerol, 16% (v/v) 0.625 M Tris pH 6.8, 4% (w/v) SDS, 0.0025% (w/v) bromophenol blue and 10% (v/v) β-mecaptoethanol). Protein samples (50 µg) were separated by molecular weight on 5–20% SDS-PAGE gels in 1x Tris/Glycine/SDS buffer (Biorad) and transferred overnight at 4°C to PVDF membranes (Amersham Bioscience). Equal loading of samples was confirmed by Ponceau S staining. Membranes were subsequently blocked with 5% (w/v) bovine serum albumin in 1x tris-buffered saline for 1 h, followed by incubation overnight at 4°C with a primary polyclonal rabbit antibody (all sourced from Cell Signaling Technology Inc., Danvers, USA) for either AKT (Cat No. #9272), AKT Ser^473^ (#4058), p70 S6K (#9202), p70 S6K Thr^421^/Ser^424^ (#9204) or 4E-BP1 Thr^37^/Thr^46^ (#9459). While, phosphorylation of Thr^37^ and Thr^46^ by mTOR does not prevent the binding of 4E-BP1 to eIF4E, it is thought to be a necessary prerequisite for subsequent phosphorylation at Ser^65^ and Thr^70^; as such, the phosphorylation state of 4E-BP1 is considered a good surrogate measure of translation initiation. This was followed by incubation with peroxidase-conjugated donkey anti-rabbit for 1 h (Amersham Biosciences), and subsequently visualised by enhanced chemiluminescence plus (Amersham Biosciences). Developed films were digitised, and band densities determined using GeneTools software (Syngene, Cambridge, UK).

### Statistical analysis

All data reported are means±S.E.M. For within-group analysis of HR and BP data, a nonparametric equivalent of analysis of variance allowing for multiple comparisons was utilised (Friedman's test). Where temporal measures of mRNA and protein levels have been made, the effects of time, treatment and time-treatment interaction were determined by two-way ANOVA. For all other analysis, or when differences reached statistical significance by two-way ANOVA, separate one-way ANOVA were performed and, where appropriate, the LSD post-hoc test used to locate any significant differences between time points. Significance was accepted at the 5% level.

## Results

### Heart rate and mean arterial pressure

**Figure 1:**
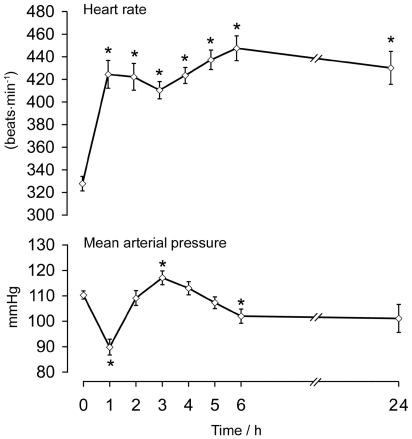
Heart rate and mean arterial pressure during continuous LPS infusion. Values are mean±SEM; n = 6 animals. * significantly different from baseline (P<0.05).

The LPS infusion resulted in early (1 h) and pronounced tachycardia which persisted for the length of the LPS infusion (Fig. 1), in addition to a biphasic fall in mean arterial pressure. The speed of onset and degree of tachycardia and hypotension were consistent with previous data obtained under similar conditions [24].

### Skeletal muscle and myocardial protein content

**Figure 2:**
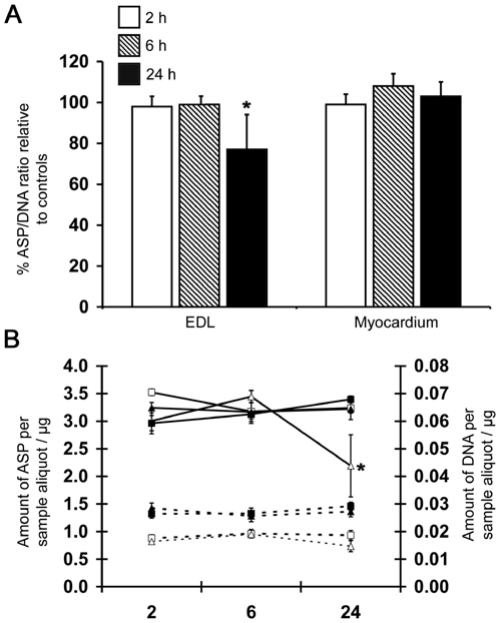
Protein to DNA ratio in muscle and myocardium following LPS infusion. A) ASP to DNA ratio following 2 h, 6 h and 24 h LPS infusion expressed as a percentage of treatment group relative to control groups. Values represent mean±SEM. n = 5–8 per group. B) Quantity of alkaline soluble proteins and DNA in a 20 µl aliquot of muscle extract. Solid lines indicate alkaline soluble proteins; dashed lines, DNA; (□) mean of values from EDL of saline treated animals; (Δ) EDL/LPS; (▪) myocardium/saline and (▴) myocardium/LPS. By two-way ANOVA, significant differences were observed for main effects of treatment (P = 0.02) and time (P = 0.03) and treatment-time interaction (P = 0.02). * indicates significantly different from control (P<0.05).

A significant reduction (23%; P<0.05) in the ASP:DNA ratio relative to controls was observed in the EDL after 24 h of LPS infusion (Fig. 2A). This appeared the result of a decline in levels of ASP in muscle extracts as opposed to a change in DNA content (Fig. 2B), indicative of a decline in muscle protein content. In contrast to skeletal muscle, there were no significant changes in ASP levels, DNA content (Fig. 2B), or the ASP:DNA ratio (Fig. 2A) of the myocardium at any time point compared to controls.

### Ubiquitin-proteasome system, TNFα and IL-6

**Figure 3:**
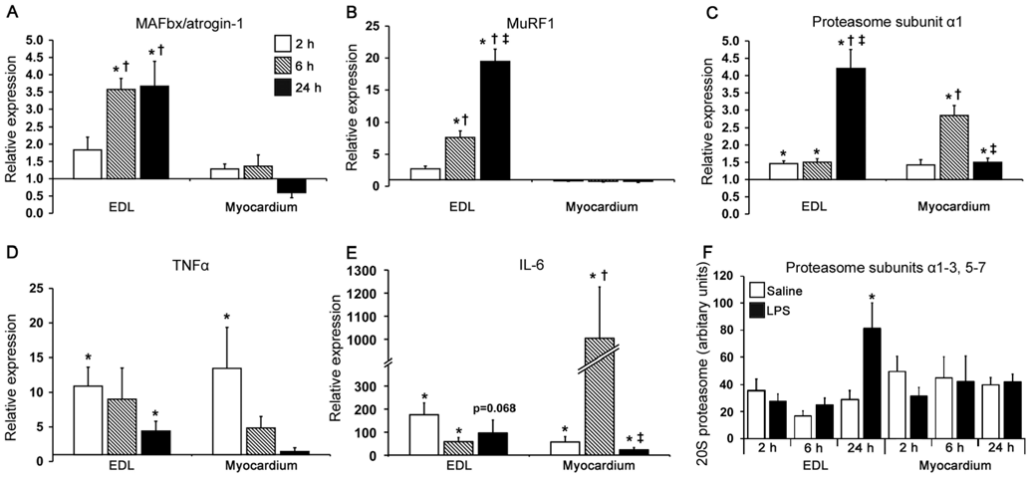
mRNA levels for proteins associated with proteolysis in muscle and myocardium following LPS infusion. Bars denote fold change in mRNA levels of A) MAFbx/atrogin-1, B) MuRF1, C) 20S proteasome subunit α1, D) TNFα and E) IL-6, compared to corresponding control values in response to either a 2 h, 6 h or 24 h i.v. LPS infusion. A value>1 indicates greater than control mRNA levels and <1 is lower then control mRNA levels. Protein levels of 20S proteasome subunits α1–3 and 5–7 were measured to confirm mRNA results for subunit alpha-1 (panel F). Values represent mean±SEM. n = 6–8 per group. By two-way ANOVA, significant differences were observed in the EDL for the main effects of treatment in all measures (P≤0.02), and for time and treatment-time interaction in all measures bar TNFα and IL-6 mRNA levels (P≤0.02). In the myocardium, significant differences for the main effect of treatment were observed in all measures bar MAFbx/atrogin-1 mRNA levels and proteasome subunit protein levels (P≤0.03). Significant differences in main effects of time and treatment-time interaction were only observed for 20S proteasome subunit α1 and IL-6 mRNA levels in the myocardium (P≤0.002). * indicates different from corresponding control (P<0.05); † different from 2 h LPS-treated muscle (P<0.05); ‡ different from 6 h LPS-treated muscle (P<0.05).

In the EDL there were markedly increased levels of MAFbx/atrogin-1 and MuRF1 mRNA within 6 h after the onset of LPS infusion (Fig. 3A & 3B respectively). Furthermore, interesting differences in the time course of change in the levels of these two ligases were observed. Thus, elevated levels of MAFbx/atrogin-1 mRNA at 6 h plateaued, whereas in contrast, MuRF1 mRNA levels were substantially elevated from the 6 h to the 24 h time-point. In addition, LPS administration resulted in the increased levels of the α1 proteasome subunit mRNA in the EDL at all examined time-points relative to control animals, but the change was particularly pronounced 24 h after the start of the LPS-infusion (Fig. 3C). Since the mRNA expression of individual subunits of the 20S proteasome can vary in response to a catabolic stimulus [30], the protein levels of subunits α1–3 and 5–7 were also determined. In agreement with findings for the α1 subunit, maximal protein levels for subunits α1–3 and 5–7 were observed in the EDL following 24 h LPS administration (Fig. 3F). While only animals subjected to 24 h LPS treatment underwent implantation of arterial catheters into the distal abdominal aorta, the reproducibility of the catabolic response to LPS in skeletal muscle to previous findings where arterial catheters were not used [3], suggest that the results observed were not due to prior surgical intervention.

In stark contrast to the EDL, the myocardium did not display altered mRNA levels for either MAFbx/atrogin-1 or MuRF1 following LPS administration at any of the three time-points examined (Fig. 3A & 3B). Interestingly, however, increased mRNA levels of the α1 proteasome subunit were detected in the myocardium 6 h and 24 h from the start of the LPS infusion (Fig. 3C), but the protein levels for multiple subunits of the 20S proteasome were unaltered (Fig. 3F).

The mRNA levels of the two cytokines, TNFα and IL-6, were examined to determine if they were in part responsible for any tissue specific changes in translation initiation or muscle protein breakdown pathways that were observed following LPS administration. Transient and significant elevations of TNFα mRNA were detected by 2 h of LPS administration in both EDL and myocardium, peaking at 11-fold and 13-fold vs controls, respectively (Figure 3D). Similarly, elevated levels of IL-6 mRNA were detected by 2 h of LPS infusion in both tissues, although of a much larger magnitude, peaking at 176-fold elevation of IL-6 mRNA in the EDL and >1000-fold in the myocardium vs controls, following 2 h and 6 h LPS administration respectively (Fig. 3E).

### Translation initiation signalling

**Figure 4:**
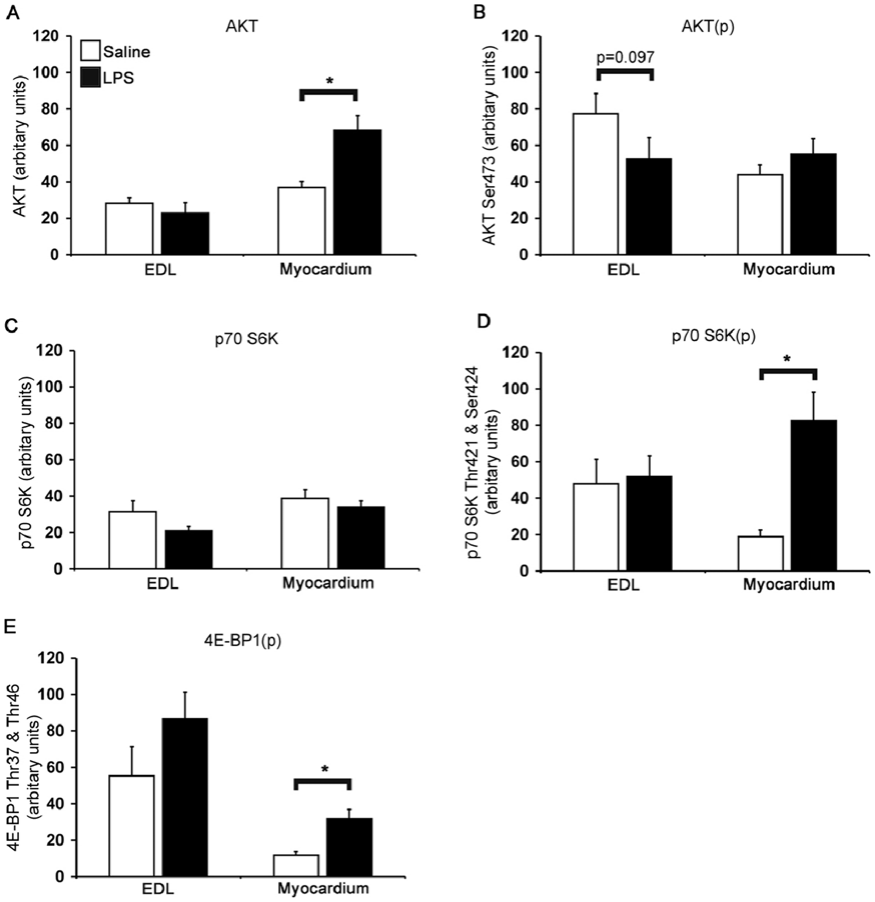
Phosphorylation and protein levels of translation initiation intermediates in muscle and myocardium following LPS infusion. Bars denote fold change in A) total AKT protein, B) phosphorylation levels of AKT residue Ser^473^, C) total p70 S6K protein, D) phosphorylation levels of p70 S6K residues Thr^421^ and Ser^424^ and E) phosphorylation levels of 4E-BP1 residues Ser^65^ and Thr^70^, in response to 24 h LPS administration relative to control animals. n = 8 per group. Values represent mean±SEM. * Indicates different from corresponding control (P<0.05).

In the EDL, the total protein level of AKT was unaffected by a 24 h LPS infusion (Fig. 4A), although a trend (P = 0.092) towards reduced phosphorylation of AKT residue Ser^473^ was detected (Fig. 4B). 24 h LPS infusion had no effect on the protein level or phosphorylation state of residues Thr^421^ and Ser^424^ of p70 S6K (Fig. 4C & 4D), nor on the phosphorylation state of Thr^37^ and Thr^46^ of 4E-BP1 (Fig. 4E), where both p70 S6K and 4E-BP1 are located downstream of AKT.

In contrast to the EDL, a significant increase in AKT protein was detected after 24 h LPS administration in the myocardium (Fig. 4A). However, LPS-induced endotoxaemia did not appear to have any significant effect on the phosphorylation state of residue Ser^473^ of AKT in the myocardium (Fig. 4B) nor on p70 S6K protein levels (Fig. 4C). However, 24 h LPS-infusion did result in a rise in the phosphorylation state of residues Thr^421^ and Ser^424^ of p70 S6K (Fig. 4D) and Thr^37^ and Thr^46^ of 4E-BP1 (Fig. 4E), indicative of a drive towards increased translation initiation in the myocardium.

## Discussion

Endotoxaemia in the rat results in a reduction in fast-twitch skeletal muscle protein content by reducing protein synthesis [2] and increasing protein breakdown [1], but it is currently unclear if a decline in myocardial protein content also occurs under these conditions, and whether any change observed is regulated by the same signalling and genomic pathways as skeletal muscle. Here we have shown myocardial protein content to be maintained at normal levels following an administration of LPS sufficient to reduce EDL protein content by 23%. Furthermore, we were able to show this disparity between the two tissues in their response to experimental endotoxaemia, is underpinned by differential responses in the processes governing muscle protein synthesis and muscle protein breakdown. The results of the present study clearly demonstrate that the events associated with UP-mediated muscle protein breakdown, as have been shown to occur in fast-twitch muscle following LPS administration [3], appear largely unaltered in the myocardium of LPS treated rats. Furthermore, the results highlight several interesting changes that occur in the mechanisms that regulate protein translation initiation in the myocardium, changes that would generally be suggestive of an enhancement of translation initiation. These observations, accompanied by the maintenance of myocardial, but not EDL protein content, suggest that the myocardium during early endotoxaemia does not experience the same catabolic fate as skeletal muscle.

While the ASP to DNA ratio was maintained in the myocardium of LPS treated animals, previous published reports detailing myocardial protein content during endotoxaemia, have reported variable findings [15], [17], [31]. It is interesting to note that where a decline in myocardial protein content has been reported, it was following the induction of endotoxaemia via the use of the cecal ligation and puncture technique in combination with the use of non-sham-operated controls [15]. O'Leary and colleagues demonstrated that performing a laporatomy, as required by the CLP technique, results in significant reductions in myocardial fractional protein synthesis rates and protein content, lasting for a minimum of 72 h post completion of the surgical procedure [17]. Thus, the variations observed between studies could be the result of methodological differences. Given the above findings, we would propose that the use of the LPS model is more appropriate to the clinical situation than CLP when considering the impact of endotoxaemia on mechanisms regulating protein turnover in rodent myocardium. However, given the sparse number of studies that have examined this issue to date, it is not possible to preclude temporal effects of endotoxaemia, induced by any one of the various means, on myocardial protein content.

Consistent with our previously reported findings [3], we observed 24 h LPS administration to result in reduced fast-twitch skeletal muscle protein content, elevated mRNA levels of MAFbx/atrogin-1 and MuRF1, and an increase in protein levels for multiple subunits of the 20S proteasome; observations that are in accordance with the reported rise in UP-mediated protein degradation during endotoxaemia in fast-twitch muscle [32]. Interestingly, while a trend towards a fall in phosphorylation of AKT residue Ser^473^ was observed, it failed to reach statistical significance (P = 0.097), and moreover, elements examined downstream of AKT (phosphorylation levels of p70 S6K and 4E-BP1) were unchanged by the LPS treatment protocol described here. This is in contrast to our previously published findings where declines in AKT Ser^473^ phosphorylation and total protein levels of AKT were reported in EDL following 24 h LPS treatment [3]. Furthermore, while levels of p70 S6K and 4E-BP1 were not examined as part of the previous study, phosphorylation of other downstream targets of AKT kinase activity, namely Foxo1 and Foxo3, were shown to be similarly reduced. Likewise, Lang and colleagues [16] have shown the bolus i.v. administration of LPS (1 mg kg^−1^) to be sufficient to reduce muscle 4E-BP1 phosphorylation after 24 h. Reasons for discrepancies between the results described here with those of previously reported findings, are unclear and difficult to discern. However, if real, reduced AKT Ser^473^ phosphorylation would appear to have had no significant impact on the two downstream translation initiation intermediates p70 S6K and 4E-BP1. The findings as reported, would suggest that derangements in UP-mediated muscle protein degradation were predominantly responsible for the observed reductions in EDL protein content, although direct measures of muscle protein synthesis and muscle protein breakdown would have been useful in providing further support of this view.

In stark contrast to the EDL, the protein content of the myocardium was unaffected by 24 h LPS administration, although an increase in phosphorylation of downstream elements of translation initiation consistent with an enhancement of muscle protein synthesis, in addition to the maintenance of MAFbx/atrogin-1 and MuRF1 mRNA at normal levels, were observed. One possible explanation for the divergent response seen between skeletal muscle and myocardium during LPS-induced endotoxaemia is the increased rate of myocardial contraction that occurs, as evidenced by the early, pronounced and sustained tachycardia seen following the start of LPS administration. Two observations described here are in support of this notion. Firstly, elevated phosphorylation levels of residues Thr^421^ and Ser^424^ for p70 S6K were observed in the myocardium of LPS-treated rats. p70 S6K indirectly promotes the translation of target oligopyrimidine (TOP) mRNAs which are almost exclusively confined to transcripts that encode for ribosomal proteins and translation factors. Contraction, at least in skeletal muscle, promotes the increased phosphorylation of p70 S6K [33] and is known to be involved in cardiac hypertrophy [34]. Secondly, we observed no change in MAFbx/atrogin-1 and MuRF1 mRNA levels in myocardial tissue following LPS administration. Jones *et al*. [29] had previously shown contraction to be sufficient to reverse limb-immobilisation-induced upregulation of the two ligases in the *vastus lateralis* of healthy human subjects. Similar observations have also been made in the gastrocnemius and soleus muscles of rats subjected to spinal cord isolation, where brief bouts of high-load isometric contractions were able to prevent the increased expression of MAFbx/atrogin-1 and MuRF1 mRNA [35]. It is possible therefore that myocardial contraction could be responsible for the maintenance of MAFbx/atrogin-1 and MuRF1 mRNA at basal levels. In further support of this notion, elevated levels of MAFbx/atrogin-1 and MuRF1 mRNA have been observed in the myocardium following chronic heart failure induced via the ligation of the left coronary artery, a condition where reduced contractile activity of the myocardium is observed [36] and, more importantly, regular exercise training has been shown to be sufficient to significantly reduce myocardial MAFbx/atrogin-1 and MuRF1 mRNA levels following experimental myocardial infarction in rats [37].

The mechanisms responsible for initiating the tissue specific changes in protein catabolism during sepsis are poorly understood. It has been widely suggested that the catabolic cytokines, TNFα and IL-6, could, in part, be responsible for triggering the catabolic events in skeletal muscle. Indeed, it has been demonstrated that both can facilitate protein catabolism in skeletal muscle and myocardium [20], [38], [39]. However, despite elevated local levels of both TNFα and IL-6 in the skeletal muscle of septic animals, their necessity for muscle atrophy to occur remains equivocal [12], [40], [41]. It is interesting to note that significantly elevated levels of TNFα and IL-6 mRNA were observed here in both the myocardium and skeletal muscle, occurring as early as 2 h after the start of LPS administration, a finding similar to that of others [14], [40], [42]. Thus, elevation of TNFα and IL-6 mRNA levels precedes increases in both MAFbx/atrogin-1 and MuRF1. However, mechanisms by which the myocardium is protected from perceived localised increases in TNFα and IL-6 remains unclear. Furthermore, reasons for the significant transient elevation of IL-6 mRNA are not possible to discern; however, the dual association of IL-6 with both contractile dysfunction [43] and cardioprotection [44] is intriguing and worthy of further investigation.

In summary, the myocardium does not experience the same reductions in protein content that are characteristically observed in skeletal muscle following continuous LPS administration. It is feasible that this is in part a consequence of the failure of the myocardium to elicit events typically associated with UP-mediated protein degradation, most notably MAFbx/atrogin-1 and MuRF1 mRNA elevation. It is also possible that events typical of enhanced protein translation initiation occur in the myocardium during early endotoxaemia, but this remains speculative with the lack of a robust reduction of AKT phosphorylation in fast-twitch muscle. The exact ‘trigger’ responsible for the tissue-specific response remains unknown. Whether myocardial contraction is responsible for mediating some of the observed differences during endotoxaemia is unclear, but is consistent with the rapid and sustained elevation in heart rate observed. Collectively, these findings represent a significant addition to our understanding of the response of skeletal and cardiac muscle to endotoxaemia and highlight possible explanations for the disparity in observations between these two tissues.
